# Selective Cholinergic Depletion in Medial Septum Leads to Impaired Long Term Potentiation and Glutamatergic Synaptic Currents in the Hippocampus

**DOI:** 10.1371/journal.pone.0031073

**Published:** 2012-02-15

**Authors:** Patrick M. Kanju, Kodeeswaran Parameshwaran, Catrina Sims-Robinson, Subramaniam Uthayathas, Eleanor M. Josephson, Nagalingam Rajakumar, Muralikrishnan Dhanasekaran, Vishnu Suppiramaniam

**Affiliations:** 1 Department of Pharmacal Sciences, Auburn University, Auburn, Alabama, United States of America; 2 Department of Neurobiology, Duke University Medical Center, Durham, North Carolina, United States of America; 3 Department of Pathobiology, Auburn University, Auburn, Alabama, United States of America; 4 Department of Neurology, University of Michigan, Ann Arbor, Michigan, United States of America; 5 Department of Neurology, School of Medicine, Emory University, Atlanta, Georgia, United States of America; 6 Department of Anatomy, Physiology and Pharmacology, Auburn University, Auburn, Alabama, United States of America; 7 Department of Anatomy and Cell Biology, The University of Western Ontario, London, Ontario, Canada; Centre National de la Recherche Scientifique, University of Bordeaux, France

## Abstract

Cholinergic depletion in the medial septum (MS) is associated with impaired hippocampal-dependent learning and memory. Here we investigated whether long term potentiation (LTP) and synaptic currents, mediated by alpha-amino-3-hydroxy-5-methyl-isoxazole-4-propionate (AMPA) and *N*-methyl-D-aspartate (NMDA) receptors in the CA1 hippocampal region, are affected following cholinergic lesions of the MS. Stereotaxic intra-medioseptal infusions of a selective immunotoxin, 192-saporin, against cholinergic neurons or sterile saline were made in adult rats. Four days after infusions, hippocampal slices were made and LTP, whole cell, and single channel (AMPA or NMDA receptor) currents were recorded. Results demonstrated impairment in the induction and expression of LTP in lesioned rats. Lesioned rats also showed decreases in synaptic currents from CA1 pyramidal cells and synaptosomal single channels of AMPA and NMDA receptors. Our results suggest that MS cholinergic afferents modulate LTP and glutamatergic currents in the CA1 region of the hippocampus, providing a potential synaptic mechanism for the learning and memory deficits observed in the rodent model of selective MS cholinergic lesioning.

## Introduction

The hippocampus is one of the major brain regions implicated in learning and memory. Excitatory neurotransmission in the hippocampus has been shown to be critical for synaptic plasticity including long term potentiation (LTP), a physiological correlate of memory [1], [2], [3]. The ionotropic glutamate receptor subtypes, alpha-amino-3-hydroxy-5-methyl-isoxazole-4-propionate (AMPA) and *N*-methyl-D-aspartate (NMDA) receptors, are the major mediators of excitatory neurotransmission and plasticity in the hippocampus [4], [5], [6]. Regulated enhancement of biophysical properties of these receptors is implicated in LTP. [4], [7], [8]. Glutamate receptor function is modulated by other neurotransmitters such as acetylcholine. The hippocampus receives major cholinergic inputs from medial septum (MS) and these cholinergic neurons regulate hippocampus-dependent learning and memory tasks [9], [10], [11], [12], [13], [14], [15], [16]. In addition, cholinergic receptors in the MS and hippocampus have been shown to modulate LTP [17], [18], [19], [20], [21], [22]. Previous studies demonstrated that memory deficits in neurodegenerative diseases are often characterized by decreased cholinergic terminals in the hippocampus [10], [11], [23], [24], [25], which further highlights the role of cholinergic receptors in memory. Overall, these findings suggest that cholinergic transmission plays an important role in hippocampal-dependent synaptic plasticity and memory tasks.

Evidence indicates that non-selective lesioning of MS impairs performance on a variety of hippocampal-dependent spatial tasks [26], [27], [28], [29], [30], [31]. On the contrary, selective cholinergic lesioning of the MS following stereotaxic infusions of immunotoxin 192-saporin, which produces significant loss of cholinergic function in the hippocampus, results in normal performance on learning and memory tasks [6], [32], [33], [34], [35], [36], [37], [38], [39]. Other studies report mild or transient impairment in memory [40], more errors in certain memory tasks [41], [42], impaired learning in T-maze tasks [43], [44], deficits in spatial strategies and navigation in spatial memory tasks [45], [46], dose-dependent deficits in working memory [47], and increasing errors with task difficulty in radial arm maze task [48], following selective lesions of cholinergic neurons in the MS. Collectively, these reports suggest that selective cholinergic lesions of the MS affect some aspects of hippocampal-dependent learning and memory tasks. Functional analyses of the outcomes of MS cholinergic lesioning, particularly in the context of synaptic plasticity in the hippocampus, are scant. Thus, in the present study we evaluated whether selective cholinergic lesioning of MS results in alterations in LTP in the CA3-CA1 synapses of the hippocampus and affects synaptic AMPA and NMDA receptor functions. Our results reveal reductions in both AMPA and NMDA receptor currents, suggesting that selective cholinergic lesions of the MS affect glutamatergic transmission in the hippocampus leading to impaired learning and memory.

## Results

### Diminished LTP in CA3-CA1 synapses in the hippocampus of rats with selective MS cholinergic lesions

**Figure 1:**
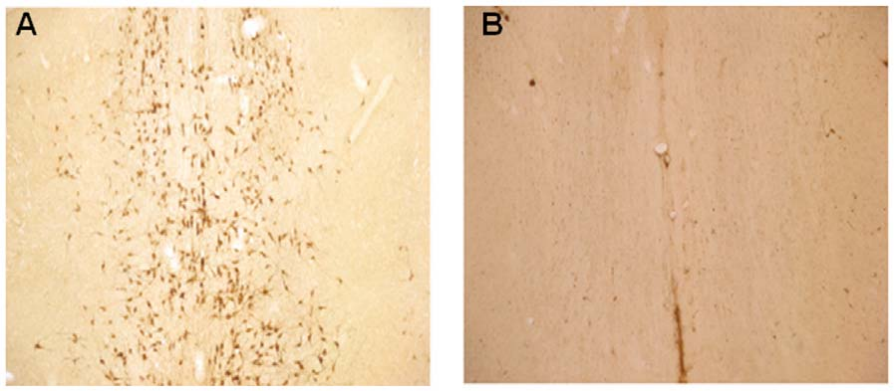
Infusion of 192-saporin results in lesioning of cholinergic neurons. Photomicrographs (50× magnification) of MS region immunostained with anti-ChAT antibody in (A) a saline infused rat and (B) in a rat infused with 192-saporin.

**Figure 2:**
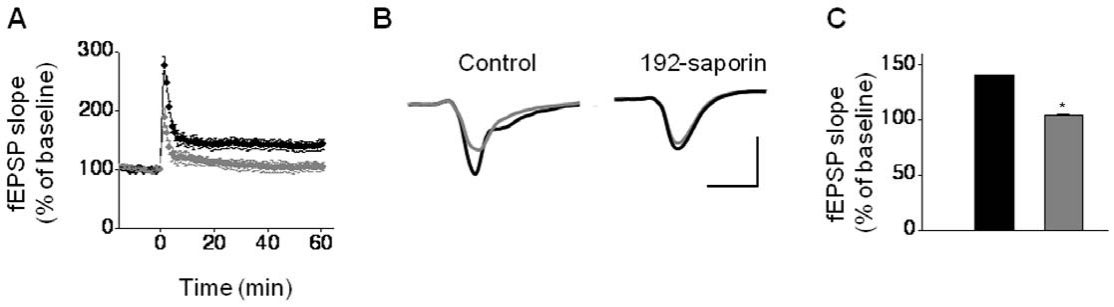
Impairment of LTP in 192-saporin infused rats. (A) Summary data for experiments in which LTP was induced by TBS and measured at 55–60 min after TBS. LTP in 192-saporin infuced rats (gray circles) was reduced compared to the saline infused controls (black circles). (B) Sample traces depicting LTP in saline infused control rats and lack of LTP in 192-saporin infused rats. Traces with gray lines represent those collected prior to TBS, during baseline recording, and the traces with black lines represent those taken 55–60 min after TBS. Calibration: 1 mV, 20 ms. (c) Bar chart showing drastic reduction of LTP in 192-saporin infused rats (gray) compared to the saline infused controls (black).

Prior to electrophysiological studies, in separate rats, we performed immunohistochemical studies to confirm that the dose, infusion site, and experimental time point resulted in a reliable and efficient cholinergic lesioning in the MS. Four to six days following infusions, we found reduced anti-choline acetyltransferase (ChAT) immunoreactive neurons, indicative of profound loss of cholinergic neurons in the 192-saporin infused rats compared to the saline infused controls (Fig. 1). This finding suggests that the 192-saporin infusion paradigm efficiently accomplished cholinergic lesioning of the MS. Previous studies reported mild, transient, or some aspects of memory deficits in rodents subjected to selective cholinergic lesioning with 192-saporin. LTP is an accepted cellular model of memory; however, it is not known whether targeted and selective cholinergic lesioning of MS alters LTP. Therefore, we evaluated LTP 4–6 days following MS lesioning. LTP was induced in CA3-CA1 synapses by theta burst stimulation (TBS) in saline infused control rats (Fig. 2; 140.75±2.35%). LTP was considerably reduced in lesioned rats (Fig. 2; 104.37±1.18%; F = 18.28, *P*<0.05, n = 6). There was no difference in LTP of saline infused animals compared to rats that underwent surgery without any infusion (data not shown). Collectively, with the accumulating evidence that acetylcholine (ACh) receptors play a role in regulating LTP in the hippocampus, our results indicate depletion of cholinergic terminals in the MS impairs LTP in the hippocampus.

**Figure 3:**
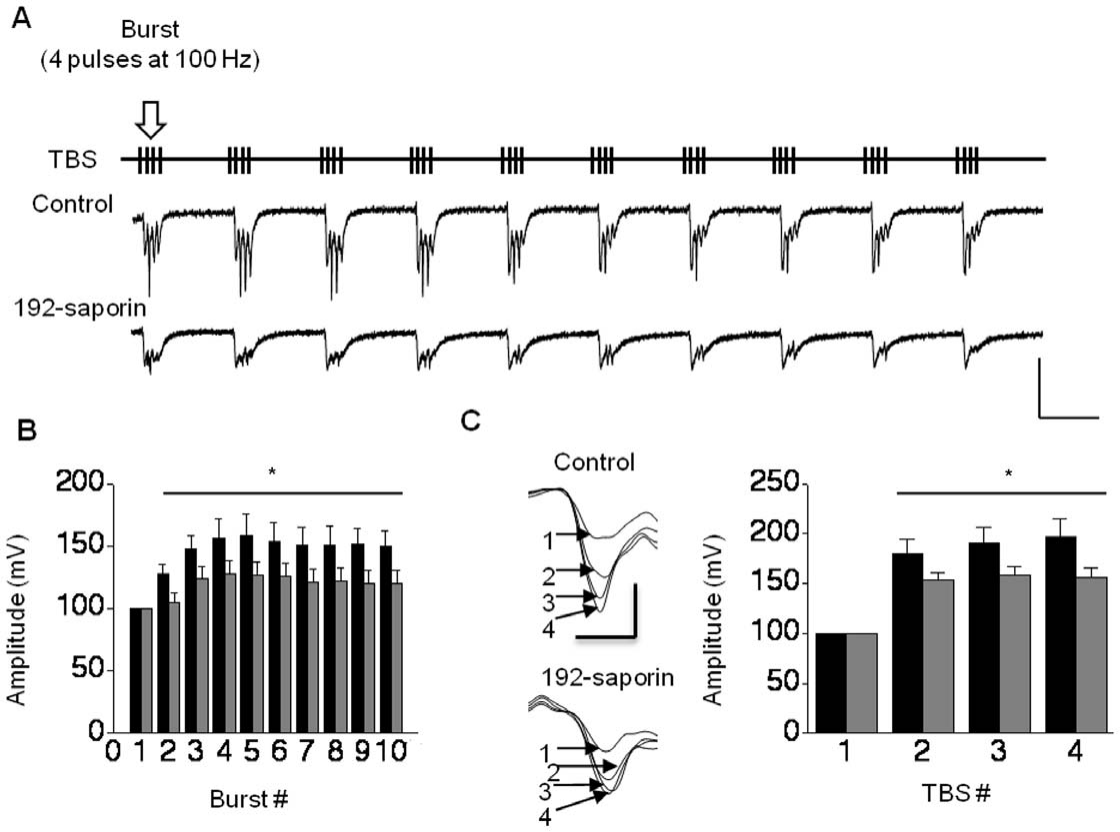
Reduction in the TBS facilitation in 192-saporin infused rats. (A) TBS stimulation protocol and samples of TBS induced traces from saline infused control and 192-saporin infused rats. (B) Bar chart exhibiting reduction of facilitation within the first TBS in 192-saporin infused rats (gray) compared to the controls (black); Calibration: 4 mV, 200 ms. (C) Facilitation of between TBS potentiation was impaired in 192-saporin infused rats (gray bars) compared to the saline infused controls (black bars) as shown by the sample traces and the bar chart. Sample traces are the fEPSPs in response to the first pulse in the first TBS. Calibration: 2 mV, 20 ms.

Having identified LTP deficits in the hippocampus of lesioned rats, we next resorted to determine which steps in LTP production were negatively impacted. The form of LTP we studied is mediated by the synaptic activation of NMDA receptors [25], [49]. Pharmacological blockade of NMDA receptors reduces TBS responses, indicating that activation of these receptors contribute to the membrane potential during TBS [50]. Therefore, we studied whether TBS responses are modified in lesioned rats. We first analyzed the within TBS facilitation by normalizing the slopes of field excitatory postsynaptic potentials (fEPSPs) with the slope of the first fEPSP. When these two sets of data were compared slices from lesioned rats showed reduced potentiation compared to the controls (Fig. 3B; F = 3.24, *P*<0.05, n = 6). We then evaluated whether subsequent TBS resulted in enhanced potentiation by normalizing the first pulse fEPSPs from 2^nd^, 3^rd^ and 4^th^ TBS to that of the 1^st^ TBS. Our results showed facilitation with each subsequent TBS in both control and lesioned rats. However, TBS facilitation in lesioned rats was lower than that of controls (Fig. 3C; F = 2.63, *P*<0.05, n = 6). These findings suggest that synaptic potentiation of NMDA receptors during TBS may be diminished in lesioned rats.

### Selective cholinergic lesions reduce AMPA receptor mediated whole cell currents in CA1 pyramidal neurons

**Figure 4:**
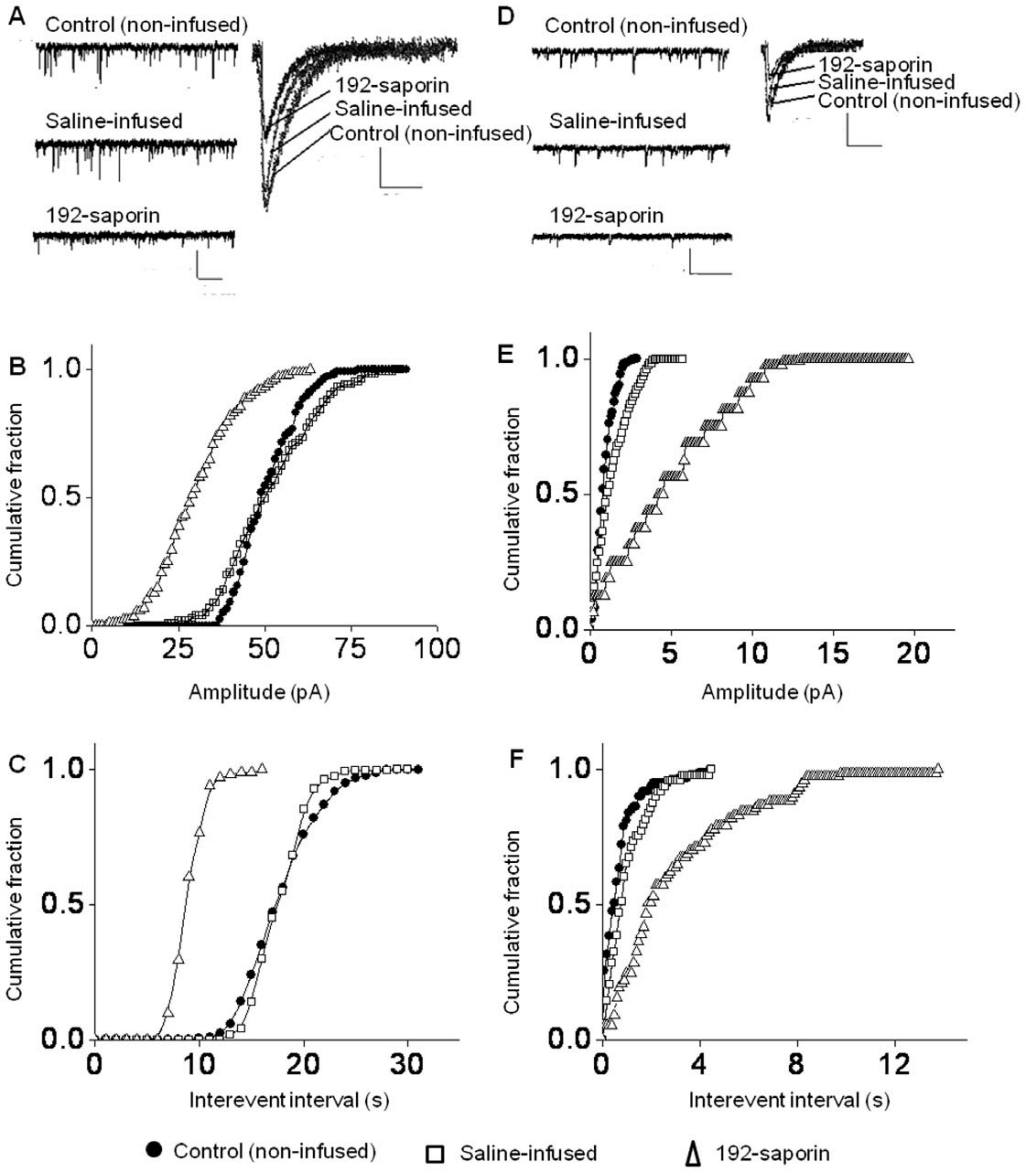
Inhibition of MS cholinergic pathway decreases the AMPA receptor activity in the CA1 pyramidal neurons of the hippocampus. (A) Representatives of AMPA receptor mediated sEPSC traces in controls and 192-saporin infused rats recorded at −65 mV membrane potential; Calibration: 40 pA, 1 s. The adjacent average traces depict the reduction in the amplitude in the MS cholinergic lesioned rats compared to control rats, which show no difference in amplitude; Calibration: 15 pA, 30 ms. (B) Cumulative fraction plot of sEPSC amplitude from the composite data shows the shift of 192-saporin curve to the left from the controls, indicating reductions in amplitude. (C) Cumulative fraction plots of sEPSC interevent intervals exhibiting increased values for 192-saporin infused rats suggesting decreased frequency. (D) Representative traces of AMPA receptor mediated mEPSCs for the three groups (Calibration: 10 pA, 1 s) and the mEPSCs (Calibration: 10 pA, 30 ms) to show reduced amplitude in the 192-saporin infused rats. (E) Cumulative fraction plots for amplitude and (F) interevent interval exhibiting reduced amplitude and frequency.

ACh is reported to be an important presynaptic modulator of synaptic transmission in glutamatergic synapses in the hippocampus [51], [52]. In CA3-CA1 synapses most of the fEPSP transmission is mediated by AMPA receptors. During LTP synapses undergo enhanced recruitment of additional AMPA receptors to the postsynaptic sites [2], [3], [53]. Since LTP was impaired in lesioned rats, we studied whether synaptic currents through AMPA receptors in the CA1 pyramidal neurons were reduced following cholinergic lesions. We recorded action potential independent miniature excitatory postsynaptic currents (mEPSCs) as well as spontaneous excitatory postsynaptic currents (sEPSCs), which occur without the inhibition of Na^2+^ channels. AMPAR-sEPSCs were recorded in the presence of 2-amino-5-phosphonovalerate (APV) (40 µM) and in the absence of tetrodotoxin (TTX) to allow action potential-driven presynaptic stimulation. Results showed that the amplitudes and frequencies of the AMPAR-sEPSCs from the saline infused and non-infused control rats were not different (Fig. 4A; Table 1; F = 0.87, n = 6, *P*>0.05). The 192-saporin infused rats showed reductions in both the amplitude and frequency of AMPAR-sEPSCs (Fig. 4A; Table 1; F = 7.38, 9.04, n = 6, *P*<0.01). Cumulative fraction histograms for amplitude were constructed for the three data sets. For amplitude the curve of 192-saporin lesioned animals was left of the two control groups indicating a reduction in amplitude (Fig. 4B). A similar shift, but to the right of control data, was observed for lesioned animals when cumulative frequency curves were constructed for inter-event intervals (Fig. 4C). This indicates that an increase in inter-event intervals (i.e. decrease in frequency) occurs in the lesioned rats.

**Table 1:** Amplitude and frequency of whole cell currents mediated by AMPA and NMDA receptors.

	Amplitude (pA)	Frequency (Hz)
*AMPAR-sEPSC:*		
Control (saline infused)	45.6±4.2	8.2±2.5
Control (not infused)	44.1±5.7	7.8±2.9
192-saporin	24.6±3.7#	2.3±0.8#
*AMPAR-mEPSC:*		
Control (saline infused)	18.7±3.2	3.6±1.2
Control (not infused)	18.4±3.6	3.2±1.4
192-saporin	8.5±2.6*	0.6±0.3*
*NMDAR-sEPSC:*		
Control (saline infused)	22.3±2.6	7.3±2.7
Control (not infused)	21.5±1.9	7.1±2.4
192-saporin	11.3±2.6#	2.8±0.9#
*NMDAR-mEPSC:*		
Control (saline infused)	11.8±1.3	2.1±1.3
Control (not infused)	12.4±1.7	2.3±1.1
192-saporin	5.8±1.6#	0.19±0.2#

*P<0.05,

# *P*<0.01.

Similar results were found when AMPAR-mEPSCs were analyzed. Both the frequency and amplitude were reduced in slices from rats subjected to MS cholinergic lesions (Fig. 4D; Table 1; F = 4.57, 17.93, n = 6, *P*<0.05). The mean amplitudes and frequencies of the AMPAR-mEPSC for saline infused and non-infused control rats were not different. Complete inhibition of mEPSCs by 6-cyano-7-nitroquinoxaline-2, 3-dione (CNQX) application (10 µM) indicated that recorded currents were mediated by AMPA receptors (data not shown). These results demonstrate a decline in AMPA receptor mediated synaptic currents in primary neurons of the CA1 region of the hippocampus in lesioned rats.

### Cholinergic lesions are associated with reduced AMPA receptor single channel properties

**Figure 5:**
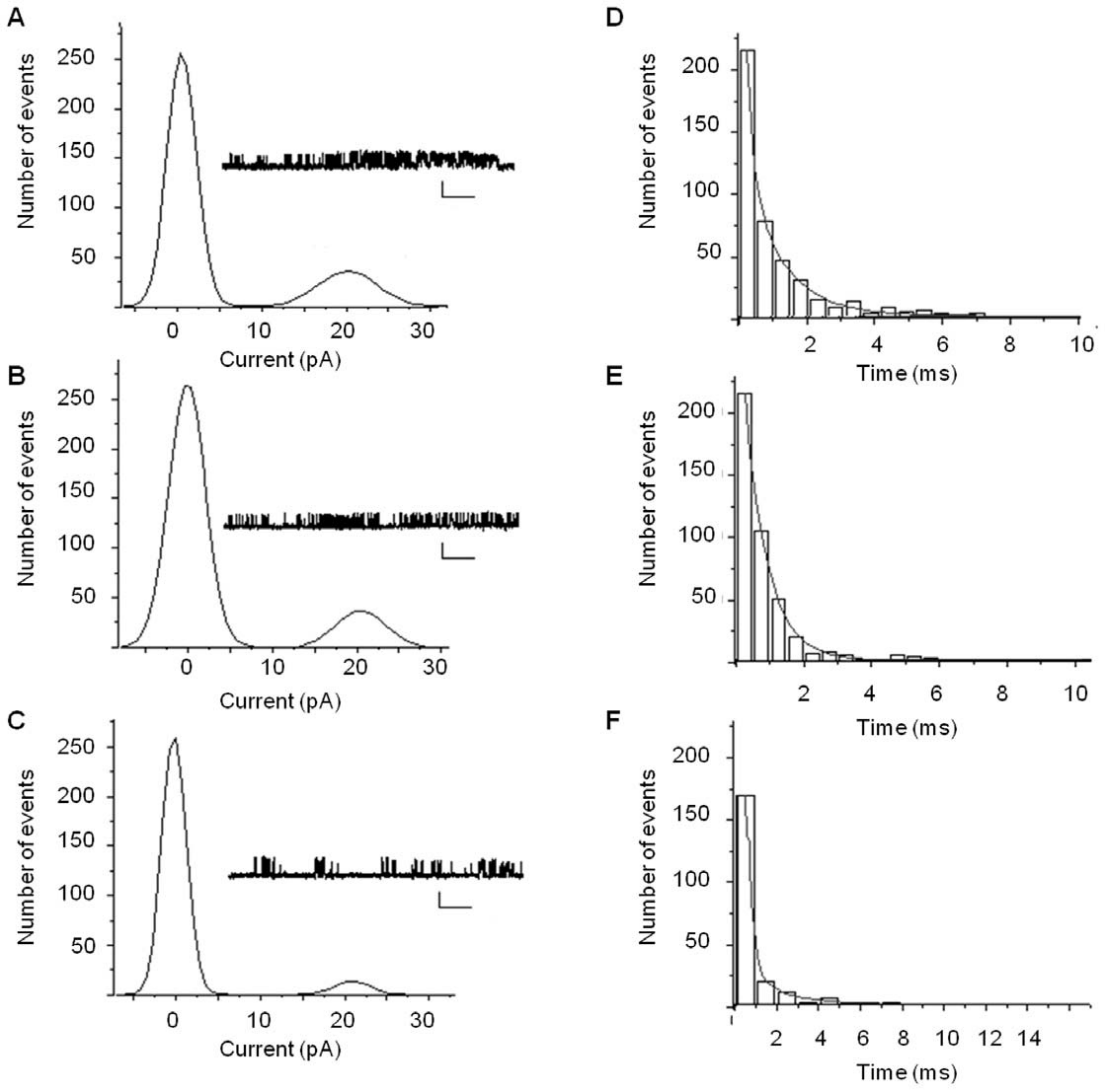
Ablation of MS cholinergic pathway alters the single channel properties of AMPA receptors. Sample traces of single channel recordings and respective current amplitude histograms for (A) non-infused, (B) saline infused, and (C) 192-saporin infused rats (Calibration: each 3 pA, 200 ms). Dwell open time histograms of (D) non-infused, (E) saline infused, and (F) 192-saporin infused rats were fitted by two exponential fittings. The AMPA elicited currents were completely blocked by the addition of CNQX (data not shown).

Channel properties of single synaptic AMPA and NMDA receptors may determine the amplitude and time course of glutamatergic synaptic transmission [54], [55]. Furthermore, AMPA receptor channel properties influence LTP. Therefore, we studied the single channel properties of AMPA receptors in biochemically isolated synaptosomes using tip-dip lipid bilayer reconstitution technique [56]. These experiments were designed to determine whether the single channel properties of synaptic receptors were altered following MS cholinergic lesions. Single channel current amplitude histograms were constructed for the two controls and the lesioned groups (Fig. 5). The probability of channel opening (Po) was not different between the two control groups (Fig. 5A and B; Table 2; F = 1.05, *P*>0.05, n = 10). Data from 192-saporin lesioned rats showed a reduction in Po that is markedly different from the control groups (Fig. 5C; Table 2; F = 8.71, *P*<0.01, n = 11). We also analyzed the dwell times of channel open and closure states and fitted the frequency histograms with the exponential decay method (Fig. 5D–F). The channel open times (τ_O_), which were best fitted with two terms, were not different between the two controls (Table 2; F = 0.79, 2.77, *P*>0.05, n = 10). The 192-saporin lesioned rats showed reductions in both of the dwell open time values (Table 2; F = 6.07, 11.39, *P*<0.01, n = 11). Another remarkable alteration in single channel property between 192-saporin infused and control rats was the run-down of single channel burst activity in the lesioned rats. The number of bursts were decreased and interburst duration was increased in 192-saporin infused rats compared to the controls (Table 2; F = 17.49, *P*<0.01, n = 10). Taken together, the data suggests that altered AMPA-EPSCs in saporin infused slices are at least, in part, due to the modified single channel properties of synaptic AMPA receptors.

**Table 2:** Burst analysis of AMPA and NMDA receptor single channel currents in synaptosomes.

	Probability of opening	Open time (τ_O1_; ms)	Open time (τ_O2_; ms)	Number of bursts	Interburst duration (ms)
*AMPA receptor currents:*					
Control (saline infused)	0.20±0.03	1.69±0.21	6.69±0.68	84±9	0.37±0.23
Control (not infused)	0.22±0.02	2.43±0.21	6.57±0.18	91±5	0.41±0.24
192-saporin	0.06±0.03#	0.26±0.41#	2.43±0.87#	16±3#	19.80±3.54#
*NMDA receptor currents:*					
Control (saline infused)	0.22±0.05	2.58±0.73	8.16±2.46	123±11	0.30±0.25
Control (not infused)	0.23±0.02	2.75±0.31	8.71±2.06	132±15	0.25±0.13
192-saporin	0.09±0.02#	0.72±0.18#	3.82±1.09#	12±6*	14.87±3.28*

*P<0.05,

# *P*<0.01.

### NMDA receptor mediated whole cell currents were decreased following cholinergic lesions

**Figure 6:**
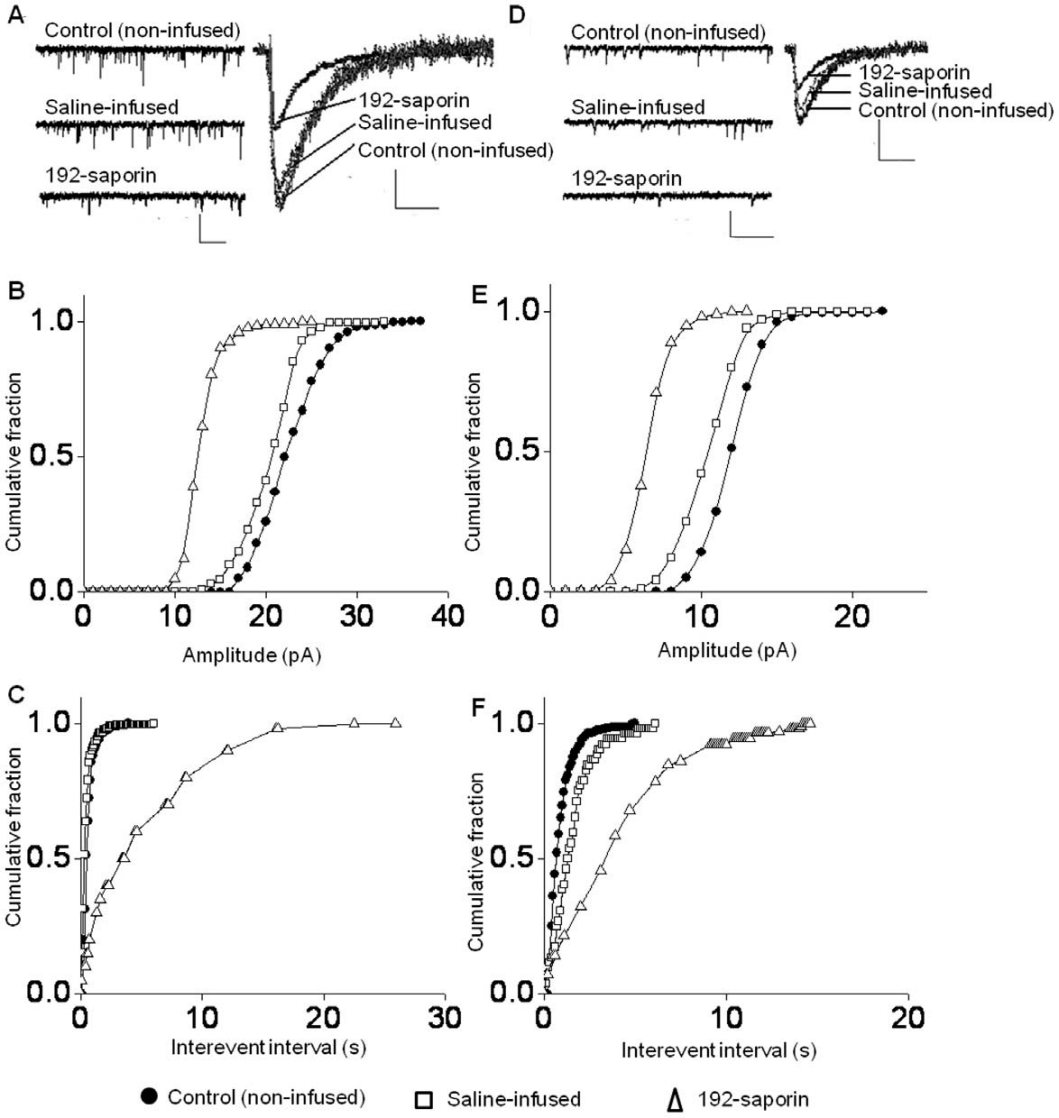
Lesioning of MS cholinergic pathway decreases the NMDA receptor mediated currents in the CA1 pyramidal neurons. (A) Representatives of NMDA receptor mediated sEPSC traces recorded at −40 mV membrane potential shows reduction of the frequency in the 192-saporin infused rats compared to the controls (Calibration: 40 pA, 1 s). The adjoining average traces of sEPSCs exhibit the reduction in amplitude in the 192-saporin infused rats (Calibration: 15 pA, 30 ms). Cumulative fraction curves of (B) amplitude and (C) interevent interval showing reduction in amplitude and frequency in the 192-saporin infused rats. (D) Representative segments of NMDA mediated mEPSCs (Calibration: 10 pA, 1 s) and average mEPSCs (Calibration: 10 pA, 30 ms) depicts reduced amplitude and frequency in the 192-saporin infused rats compared to the two controls. Cumulative fraction curves for (E) amplitude and (F) interevent interval of mEPSCs.

The NMDA receptors, the major glutamate receptor subtype with high Ca^2+^ permeability, play a vital role in the induction phase of LTP in the CA1 region [1], [57]. Our results suggest that LTP induction is impaired in rats subjected to MS cholinergic lesions. Therefore, we investigated the effect of 192-saporin induced cholinergic lesioning on the functional properties of NMDAR-sEPSCs and NMDAR-mEPSCs. The average amplitudes of sEPSCs in the saline infused and non-infused controls were not different; likewise, the frequencies between these two groups were not different. Hence, there were no difference between the controls (Fig. 6A; Table 1; F = 1.07, *P*>0.05, n = 9). Analysis of hippocampal slices from 192-saporin infused rats revealed decreases in both the frequency and the amplitude of NMDAR-sEPSCs (Fig. 6A; Table 1; F = 8.35, 12.82, *P*<0.01, n = 7). Cumulative fraction plots for amplitude (Fig. 6B) and inter-event interval (Fig. 6c) from all experimental data indicate reductions in both amplitude and frequency of NMDAR-sEPSCs in the 192-saporin infused rats.

Analysis of the NMDAR-mEPSCs indicated a reduction in the average amplitude and frequency in the 192-saporin infused rats (Fig. 6D). There was no difference in the amplitude or frequency of NMDAR-mEPSCs from the two control groups (Fig. 6; Table 1; F = 1.21, 1.58, *P*>0.05, n = 9). The NMDAR-mEPSCs recorded in hippocampal slices from 192-saporin infused rats showed decreases in both amplitude and frequency (Fig. 6; Table 1; F = 8.04, 10.96, *P*<0.01, n = 9). Cumulative fraction plots for amplitude (Fig. 6E) and inter-event interval (Fig. 6f) depicts reductions in both amplitude and frequency of NMDAR-mEPSCs in the 192-saporin infused animals. In summary, these results indicate reduction of NMDAR mediated synaptic currents associated with cholinergic lesioning of the MS.

### Cholinergic lesioning impairs single channel properties of NMDA receptors in isolated synaptosomes

**Figure 7:**
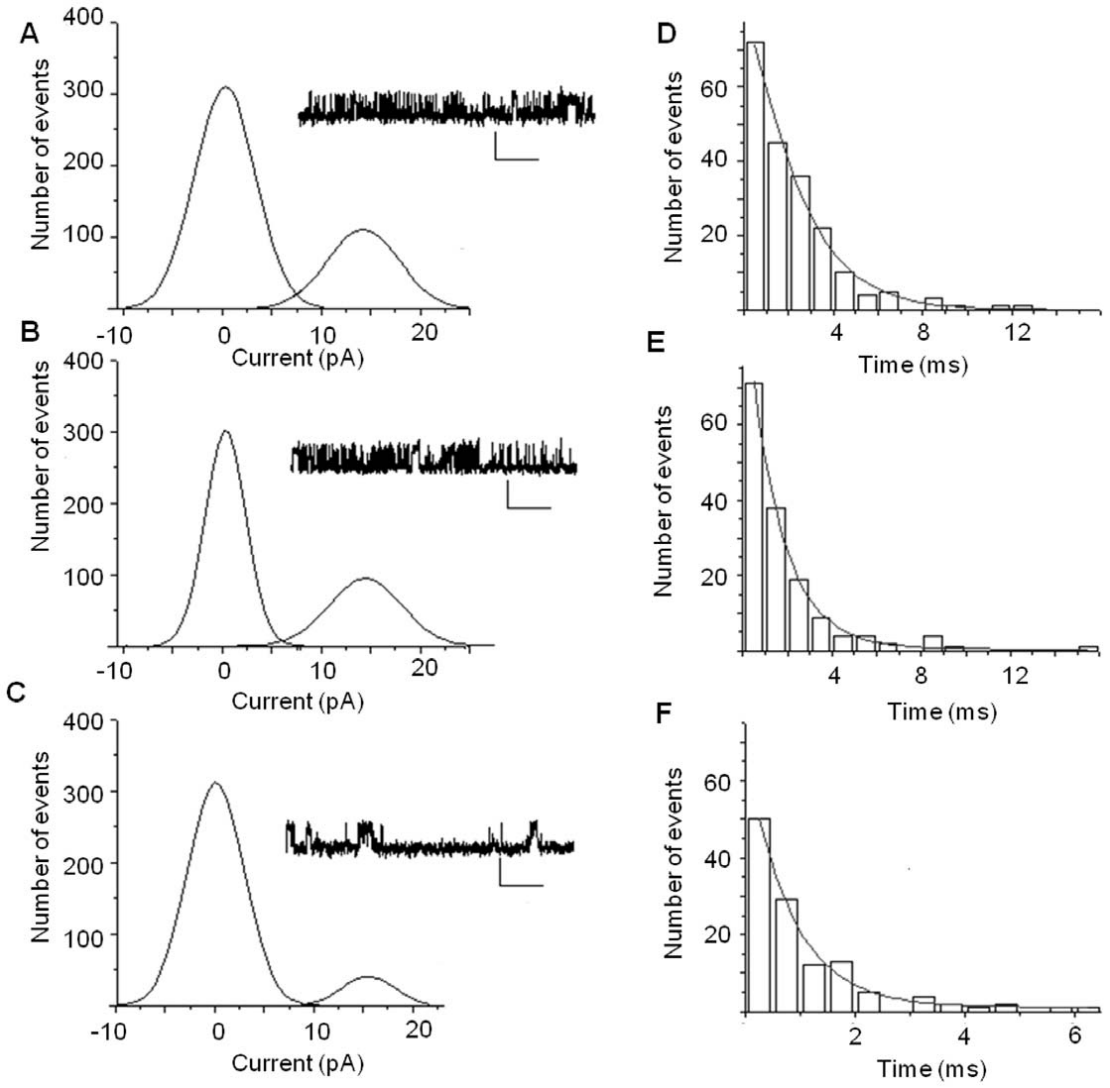
Cholinergic depletion in the MS leads to alterations in NMDA elicited synaptic single channel currents. Sample traces and respective current amplitude histograms of (A) non-infused control rats, (B) saline infused rats, and (C) 192-saporin infused rats show that channel open peak (right peaks in each histogram) in the 192-saporin data is reduced demonstrating a decrease in open probability (Calibration: each 4 pA, 200 ms). Dwell open time histograms of NMDA currents were best fitted with two terms for (D) non-infused, (E) saline infused, and (F) 192 saporin infused data. The NMDA elicited currents were confirmed by addition of APV to the extracellular solution to completely block these currents (Data not shown).

Single channel currents of synaptic NMDA receptors exhibited no difference (Fig. 7; Table 2; F = 0.88, 1.29, *P*>0.05; n = 10) in either the Po or the τ_O_ values between the non-infused and saline-infused controls. The Po and the τ_O_ values of 192-saporin infused animals were reduced compared to controls (Fig. 7; Table 2; F = 7.98, 13.26, *P*<0.01, n = 11). The bursting activity of synaptic single channel NMDA elicited currents was also reduced. The number of bursts was drastically reduced in lesioned animals while the inter-burst duration was prolonged in lesioned animals (Table 2; F = 8.53, 11.07, *P*<0.01, n = 11). These results suggest that the single channel properties of synaptic NMDA receptors are decreased, which may contribute to the reduction in whole cell currents.

## Discussion

The current study investigated LTP and the functional properties of synaptic AMPA and NMDA receptors in the hippocampus following immune-lesioning of the MS cholinergic neurons. Specifically, the intrinsic electrophysiological properties of AMPA and NMDA receptors were studied in both whole cell slice recordings and single channel recordings from isolated synaptosomes. Recordings were performed in hippocampal CA1 region 4–6 days following medial septal lesioning with 192-saporin. The coordinates and the dose of 192-saporin used in this study are suitable for targeting the medial septum and vertical limb of the diagonal band cholinergic terminals and low enough to avoid non-specific damage [58]. As established in previous reports, the 192-saporin administration resulted in selective cholinergic lesioning in female rats [36], [59], [60]. The experimental time point was selected because cholinergic neurons show significant neurodegeneration and prior transient declines in learning and memory occur in this animal model [43], [44], [45].

Several studies demonstrated that various forms of cognitive tasks are controlled by the interaction of both glutamatergic and cholinergic systems. Several studies have established through pharmacological manipulations that interactive modulation between the cholinergic and glutamatergic neurotransmitters systems represents a critical mechanism in different cognitive functions [61], [62], [63], [64], [65], [66], [67]. Furthermore, interaction of these neurotransmitter systems in the hippocampus has also been reported to regulate cognitive tasks [68], [69], [70], [71]. Physiologically, interactions between cholinergic and glutamatergic systems can control forms of synaptic plasticity [72], [73] that may regulate cognitive processes [74], [75], [76]. More specifically septohippocampal afferents release ACh and generate the theta rhythm that can influence memory. Prolonged activation of muscarinic receptors may enhance glutamatergic synaptic efficacy [17], [77] implying that cholinergic activation may play a vital role in memory encoding [75]. In further support, a recent study demonstrated that activation of muscarinic receptors contributed to a form of LTP in CA1 pyramidal neurons, through intracellular uncaging of Ca^2+^ ions [78]. Other brain regions such as the visual cortex also undergo changes in plasticity and switch from LTP to long term depression (LTD) upon TBS induction, when cholinergic projections were selectively lesioned by injecting 192-saporin into the lateral ventricles of young rats [79]. Endogenously released ACh facilitated LTP in the hippocampus. Lesioning of cholinergic neurons in the MS reduces electrically evoked ACh [80], which facilitates LTP in the hippocampus [73], and may be responsible for the sharp decline in LTP. A previous report described impaired LTP induction following intraventricular infusion of the immunotoxin saporin [81]. The intraventricular administration in this study caused a more general decline in cholinergic terminal across more brain regions compared to our study. Furthermore, only MS cholinergic afferents were lesioned and only hippocampal afferents were targeted in our study, demonstrating that this region is critical for LTP in the hippocampus. Hence, these previous studies support our results that LTP is severely diminished shortly after the selective cholinergic lesioning of the MS. Since LTP is a widely accepted cellular model for memory encoding, learning and memory deficits are often reported along with diminished LTP in the hippocampus. While some studies reported no change in memory upon MS lesioning of cholinergic terminals, as emphasized in the introduction of this report, a detailed analysis of many of the reports revealed mild and/or transient decreases in learning and memory. This explains our results that LTP is decreased at an early stage following lesioning and strengthen the notion that selective cholinergic lesioning in the MS parallels a decline in learning and memory as well as LTP in the hippocampus.

During LTP, quantal size increases and silent synapses that are devoid of surface AMPA receptors may become active following the postsynaptic recruitment of AMPA receptors [36]. Results from our experiments indicate a decrease in both frequency and amplitude of mEPSCs, suggesting a decreased probability of release and quantal content. Cholinergic receptors are known for their regulatory role on glutamate receptors in the hippocampus. Developmental exposure to nicotine resulted in decreased AMPA receptor currents in the CA1 pyramidal cells [82] and enhanced NMDA currents in the developed hippocampus [83]. These findings support a mechanism where modulation of ACh receptors regulates both NMDA and AMPA receptor mediated synaptic currents in the hippocampus. Impairments in the basal, true synaptic currents mediated by AMPA and NMDA receptors may also be responsible for the decline in LTP in 192-lesioned rats. Therefore, our results along with previous reports suggest that depletion of cholinergic neurons in the MS disrupts the ACh receptor mediated regulation of AMPA and NMDA receptor currents in the CA1 region of the hippocampus.

The septohippocampal cholinergic innervations are also known to control gamma-amino butyric acid (GABA)-ergic interneurons of the CA1 field [84]. Studies show that cholinergic control of the GABAergic interneurons was abolished in 192-saporin infused rats [85], yet the GABAergic inhibitory synaptic events were unchanged in 192-saporin treated animals compared with controls [86]. The GABAergic interneurons regulate the glutamate currents in the primary pyramidal neurons in the CA1 region, however, in our experimental conditions addition of GABA_A_ blockers to the extracellular solution in both control and treated animals largely eliminated this possibility. A previous study reported that cholinergic currents in the CA1 neurons were insensitive to GABA_B_ blockers [87]. Hence, alterations in the inhibitory currents from GABAergic neurons are unlikely to be responsible for changes in AMPA and NMDA receptor function.

A novel and interesting finding of this study is the altered single channel properties of synaptic AMPA and NMDA receptors. This adds support to the modified mEPSC properties as mEPSC amplitude and single channel open probability are positively correlated [88]. The amplitude of glutamate mediated mEPSCs in the CA1 neurons are the product of the summation of single channel currents from the respective receptors, which are activated by the quantal release of glutamate from presynaptic terminals. Therefore, alterations in amplitude of EPSCs observed in lesioned hippocampi in this study are partly due to altered single channel current. It is also known that modifications in single channel properties of synaptic AMPA receptors can influence LTP [4], [7], [8]. Consistently, analysis of single channel properties of synaptic AMPA and NMDA receptors indicated significant reductions in open probabilities and open times. Therefore, apart from the strong possibility of altered quantal content, postsynaptic modifications in single channel properties also play a role in reducing the whole cell currents as well as reduction in LTP. The single channel properties of glutamate receptors are regulated by kinase mediated phosphorylation of intracellular domains [89], [90], [91], [92], [93], [94]. Interestingly, reduced cholinergic activity may result in decreased kinase activities. A recent report showed that lack of m1 muscarinic receptors resulted in reduced PKC activity [95]. Lack of cholinergic input has also been shown to decrease PKA activity [96], which is involved in the regulation of single channel properties of AMPA receptors. Therefore, it is reasonable to presume that reduced kinase activity associated with selective cholinergic lesioning resulted in altered single channel properties of glutamate receptors.

In summary, our findings provide physiological support to the existing hypothesis that septohippocampal cholinergic projections modulate learning and memory by regulating single channel properties of synaptic glutamate receptors [97], [98], [99]. Specifically, we demonstrate that septohippocampal cholinergic projections modulate hippocampal glutamatergic synaptic transmission and thereby, alter synaptic plasticity mechanisms required for learning and memory.

## Materials and Methods

### Animals, surgical Procedure, and slice preparation

Female Sprague Dawley rats weighing 230–300 g were individually caged and kept in a 12-h light/dark cycle. Rats were anesthetized (Ketamine:Xylazine, 87 mg/kg:13 mg/kg; i.p.), placed on a stereotaxic frame, a dorsal midline skin incision was made over the skull, and a small burr hole was drilled. The immunotoxin 192-saproin (50 ng in 2 µl per rat) (192 IgG Saporin, Advanced Targeting Systems, San Diego, CA) in sterile saline was infused into the MS through a Hamilton syringe (0.4 µl/min for 5 min) using the following stereotaxic coordinates: from bregma, AP +0.2 mm; DV −6.0 mm (from the skull); ML 0.0 mm [100]. Control rats received similar infusions of sterile saline. The needle was left in place for an additional 5 min to prevent backflow of solution along the needle track. A separate set of rats was subjected to the above procedure except that no infusions were made. Four to six days after surgery the rats were briefly anesthetized, decapitated, and the brains were isolated in ice cold dissection buffer containing (in mM): 85 NaCl, 2.5 KCl, 4 MgSO_4_, 0.5 CaCl_2_, 1.25 NaH_2_PO_4_, 25 NaHCO_3_, 25 glucose, 75 sucrose 0.5 ascorbate, and 2 kynurenic acid bubbled with 95%CO_2_/5%O_2_ at pH 7.4. Coronal brain slices (400 µm) with hippocampi were cut on a vibratome while immersed in the oxygenated dissection buffer. Slices were immediately transferred to an incubation chamber containing artificial cerebrospinal fluid (aCSF) consisted of (in mM): 119 NaCl, 2.5 KCl, 1.3 MgSO_4_, 2.5 CaCl_2_, 1 NaH_2_PO_4_, 26 NaHCO_3_, and 11 dextrose bubbled with 95%CO_2_/5%O_2_ at pH 7.4. For slices that were subjected to whole cell current recordings the aCSF was supplemented with 2 mM kynurenic acid during the incubation. Slices were incubated for at least 1 hour before the electrophysiological recordings. All chemicals for preparation of solutions were purchased from Sigma (St. Louis, MO). All the ion channel blockers were from Tocris (Ellisville, MO). TTX was from Sigma. Animal housing, handling, and experimentation were performed as per the protocol approved by Auburn University Institutional Animal Care and Use Committee (IACUC; PRN 2003-0454).

### Immunohistochemistry

Randomly-selected groups of rats were anesthetized with sodium pentobarbital (50 mg/kg; i.p.) and transcardially perfused with 4% paraformaldehyde (PFA) in phosphate buffered saline at pH 7.2 (PBS). Brains were kept in the fixative overnight and then transferred to 30% sucrose in PBS and stored at 4°C until use. Brain sections (16 µm) consisting MS were cut on a cryostat and were processed for immunohistochemistry. Sections were washed in cold PBS (pH 7.2) several times and endogenous peroxidase activity was quenched by incubating for 10 min at room temperature (RT) with 0.3% H_2_O_2_ in 0.01 M PBS. Non-specific labeling was blocked by incubating with 10% normal horse serum (ICN Biomedicals, Aurora, OH) in PBS for 30 min at RT. Sections were then incubated in a solution containing rabbit anti-choline acetyltransferase (ChAT) antibody (Chemicon, Temecula, CA, USA) diluted 1∶500 in 10% normal horse serum in PBS for 12 hrs at 4°C. Sections were incubated with goat anti-rabbit biotinylated secondary antibody (Vector, Burlingame, CA) diluted 1∶1,000 in 10% normal horse serum in PBS. Signal was amplified by incubation with avidin-biotin-peroxidase complex (ABC kit, Biomeda, FosterCity, CA). Labeling was visualized with 1% diaminobenzidine tetrahydrochloride (DAB) and 0.03% H_2_O_2_ in PBS. Following several washes in PBS sections were mounted onto slides for imaging.

### Extracellular recordings in slices

Slices were transferred to a submerged type recording chamber and continuously perfused with oxygenated aCSF. fEPSPs were evoked by stimulating Shaffer collateral afferents in the stratum radiatum with a bipolar electrode (MPI, Gaithersburg, MD) and recordings were made in the CA1 axons with a glass pipette filled with aCSF (1–4 MΩ). Signals were digitized, amplified and acquired using LTP program [4]. Stimulation command was provided through the LTP program and was regulated by a stimulus isolator. After recording fEPSPs for 15 min, with an amplitude that is 50% of the sub-threshold maximum, LTP was induced by delivering 5 trains of TBS separated by 20 sec. Each TBS consisted of 10 trains of bursts, each of which had 4 pulses at 100 Hz, with an inter burst interval of 200 ms. LTP was measured at 55–60 min post TBS.

### Whole cell recordings

Slices were placed in a submerged type recording chamber and continuously perfused with oxygenated aCSF. Individual CA1 pyramidal neurons were visually identified and selected utilizing a Nomarski differential interference contrast microscope with water immersion optics (Olympus BX51W). Conventional intracellular recordings from selected neurons were obtained using glass micropipettes (5–10 MΩ) that were filled with a solution containing:100 mM K-gluconate, 0.6 mM EGTA, 5 mM MgCl_2_, 2 mM ATP-Na, 0.3 mM GTP-Na and 40 mM HEPES. Intracellular recordings of mEPSCs and sEPSC were recorded with axopatch 200B amplifier (Molecular Devices, Sunnyvale, CA).

To isolate AMPA receptor mediated mEPSCs (AMPAR-mEPSC), recordings were performed in the presence of the GABA receptor antagonist picrotoxin (50 µM), Na^+^ channel blocker TTX (1 µM), and NMDA receptor antagonist APV (40 µM). The recordings of NMDA receptor mediated mEPSCs (NMDAR-mEPSC) were similar to those of AMPA except that APV was replaced with CNQX (4 µM) to block AMPA currents and the aCSF was Mg^2+^ free. To record sEPSC of both the NMDA and AMPA components the conditions mentioned above were maintained. However, the extracellular calcium∶magnesium ratio was altered to 4∶1 to exploit the release probability and to rule out quantal variability of pre-synaptic glutamate.

### Preparation of Synaptosomes

Hippocampi were rapidly dissected out from randomly selected groups of anesthetized rats and synaptosomes were prepared as previously described [101] with some modifications. Briefly, hippocampi were homogenized with modified Kreb-Henseleit (mKREBS) buffer containing 118.5 mM NaCl, 4.7 mM KCl, 1.18 mM MgSO_4_, 2.5 mM CaCl_2_, 1.18 mM KH_2_PO_4_, 24.9 mM NaHCO_3_, 10 mM dextrose, 10 mg/ml adenosine deaminase and pH was adjusted to 7.4 by bubbling with 95%/5%:O_2_/CO_2_. The mKREBS buffer was supplemented with 0.01 mg/ml leupeptin, 0.005 mg/ml pepstatin A, 0.10 mg/ml aprotinin and 5 mM Benzamide. The homogenate was diluted adequately with mKRBS buffer before being filtered through a 13 mm diameter millipore syringe filter. The filtrate was forced through a nylon filter (100 µm pore size). The pre-filtered mixture was further filtered through a 5 µm Millipore syringe filter (Millex SV) pre-wetted with mKRBS buffer. The filtrate was then spun at 1000× g for 15 min in a microfuge at 4°C. The supernatant was discarded and pellets (synaptosomes) were re-suspended in mKREBS buffer and stored at −80°C.

### Single channel recordings in synaptosomes

Single channel recordings from synaptic AMPA receptors were performed as described previously [56]. In brief, a phospholipid bilayer [made of 1,2 diphytanoyl-sn-glycero-3-phosphocholine (Avanti Polar-Lipids Inc., Alabaster, AL) in anhydrous hexane (Aldrich Chemical Co., Milwaukee, WI); 1 mg/ml], was formed by tip-dip method at the tip of a glass pipette (100 MΩ) filled with an internal solution consisted of (in mM): 110 KCl, 4 NaCl, 2 NaHCO_3_, 1 MgCl_2_, 0.1 CaCl_2_, and 2 MOPS (pH adjusted to 7.4). The extracellular bath solution (∼300 µl) contained (in mM): 125 NaCl, 5 KCl, 1.25 NaH_2_PO_4_, and 5 Tris HCl. Once a stable membrane was formed, 3–5 µl suspension of the synaptosomes was delivered to the bath solution and the ion channel specific agonist was added. Voltage was applied across the membrane to evoke single channel activity of the receptors that are reconstituted in the bilayer membrane. To record AMPA receptor currents, 290 nM AMPA was added to the bath solution which was supplemented by 50 µM APV (NMDA antagonist), 1 µM SYM2081 (kainate receptor antagonist), 100 µM Picrotoxin (GABA_A_ receptor antagonist), 2 µM TEA (potassium channel antagonist), and 1 µM TTX (sodium channel antagonist). At the end of each recording, AMPA currents were confirmed by the addition of 1 µM CNQX. NMDA receptor currents were activated by 3 µM of NMDA in the presence of 1 µM SYM2206 (AMPA receptor antagonist), 1 µM SYM2081, 100 µM picrotoxin, 2 µM TEA, and 1 µM TTX. NMDA currents were confirmed by the addition of 50 µM of APV.

Single channel currents were filtered at 2 kHz and digitized at 5 kHz (Mini-digi, Molecular Devices) with pClamp9 software (Molecular Devices) and saved in a computer. Only the data exhibiting long stretches of single channel current transition without base line drifts were chosen for quantitative analysis.

### Data analysis

The mEPSC, sEPSC, and LTP data were analyzed with one-way ANOVA. The mEPSC and sEPSC data were analyzed with Mini Analysis program (Synaptosoft, Fort Lee, NJ). The EPSC amplitude (A) was measured from the baseline with the amplitude threshold for detection of mEPSCs was set above the noise level, at four times the SD of mean noise level, and individual mEPSCs satisfying these criteria were selected manually. For single channel analysis only the traces showing stable baseline without drastic shifts and having recurrent channel activity were chosen. The single channel open probability was estimated as Po = *R*_o_/(*R*_c_+*R*_o_), where *R*_c_ and *R*_o_ represent the areas under the current-amplitude histogram corresponding to close and open states, respectively [56]. The all points-current amplitude histograms was constructed and fitted with two-term Gaussian method using Microcal Origin program (OriginLab Corp., Northampton, MA) to identify individual conductance levels of channel open and close states. The single channel conductance was computed by plotting current as a function of membrane voltage, according to the equation *g* = *I*/(*V*−*V*_o_), where *I* is the single channel current, *V* is the voltage, and *V*_o_ is the reversal potential [56]. Open and close time histograms were fitted best with the exponential probability function and variable metric method using pClamp 9.0 Program. Data were analyzed with ANOVA with Tukey's post hoc test, and presented as mean±SEM with statistical significance accepted at *P*≤0.05.

## References

[1] MacDonald JF, Jackson MF, Beazely MA (2006). Hippocampal long-term synaptic plasticity and signal amplification of NMDA receptors.. Crit Rev Neurobiol.

[2] Malenka RC (2003). Synaptic plasticity and AMPA receptor trafficking.. Ann N Y Acad Sci.

[3] Maren S, Tocco G, Standley S, Baudry M, Thompson RF (1993). Postsynaptic factors in the expression of long-term potentiation (LTP): increased glutamate receptor binding following LTP induction in vivo.. Proc Natl Acad Sci U S A.

[4] Ambros-Ingerson J, Lynch G (1993). Channel gating kinetics and synaptic efficacy: a hypothesis for expression of long-term potentiation.. Proc Natl Acad Sci U S A.

[5] Benfenati F (2007). Synaptic plasticity and the neurobiology of learning and memory.. Acta Biomed.

[6] Bizon JL, Han JS, Hudon C, Gallagher M (2003). Effects of hippocampal cholinergic deafferentation on learning strategy selection in a visible platform version of the water maze.. Hippocampus.

[7] Benke TA, Luthi A, Isaac JT, Collingridge GL (1998). Modulation of AMPA receptor unitary conductance by synaptic activity.. Nature.

[8] Holmes WR, Grover LM (2006). Quantifying the magnitude of changes in synaptic level parameters with long-term potentiation.. J Neurophysiol.

[9] Amaral DG, Kurz J (1985). An analysis of the origins of the cholinergic and noncholinergic septal projections to the hippocampal formation of the rat.. J Comp Neurol.

[10] Araujo DM, Lapchak PA, Robitaille Y, Gauthier S, Quirion R (1988). Differential alteration of various cholinergic markers in cortical and subcortical regions of human brain in Alzheimer's disease.. J Neurochem.

[11] Bartus RT, Dean RL, Beer B, Lippa AS (1982). The cholinergic hypothesis of geriatric memory dysfunction.. Science.

[12] Bartus RT, Flicker C, Dean RL, Pontecorvo M, Figueiredo JC (1985). Selective memory loss following nucleus basalis lesions: long term behavioral recovery despite persistent cholinergic deficiencies.. Pharmacol Biochem Behav.

[13] Easton A, Fitchett AE, Eacott MJ, Baxter MG (2010). Medial septal cholinergic neurons are necessary for context-place memory but not episodic-like memory.. Hippocampus.

[14] Everitt BJ, Robbins TW (1997). Central cholinergic systems and cognition.. Annu Rev Psychol.

[15] Hasselmo ME (2006). The role of acetylcholine in learning and memory.. Curr Opin Neurobiol.

[16] Navakkode S, Korte M (2011). Cooperation between cholinergic and glutamatergic receptors are essential to induce BDNF-dependent long-lasting memory storage.. Hippocampus.

[17] Auerbach JM, Segal M (1994). A novel cholinergic induction of long-term potentiation in rat hippocampus.. J Neurophysiol.

[18] Blitzer RD, Gil O, Landau EM (1990). Cholinergic stimulation enhances long-term potentiation in the CA1 region of rat hippocampus.. Neurosci Lett.

[19] Kenney JW, Gould TJ (2008). Modulation of hippocampus-dependent learning and synaptic plasticity by nicotine.. Mol Neurobiol.

[20] Mansvelder HD, Mertz M, Role LW (2009). Nicotinic modulation of synaptic transmission and plasticity in cortico-limbic circuits.. Semin Cell Dev Biol.

[21] Matsuyama S, Matsumoto A, Enomoto T, Nishizaki T (2000). Activation of nicotinic acetylcholine receptors induces long-term potentiation in vivo in the intact mouse dentate gyrus.. Eur J Neurosci.

[22] Placzek AN, Zhang TA, Dani JA (2009). Nicotinic mechanisms influencing synaptic plasticity in the hippocampus.. Acta Pharmacol Sin.

[23] Daulatzai MA (2010). Early stages of pathogenesis in memory impairment during normal senescence and Alzheimer's disease.. J Alzheimers Dis.

[24] Hernandez CM, Kayed R, Zheng H, Sweatt JD, Dineley KT (2010). Loss of alpha7 nicotinic receptors enhances beta-amyloid oligomer accumulation, exacerbating early-stage cognitive decline and septohippocampal pathology in a mouse model of Alzheimer's disease.. J Neurosci.

[25] Morgan SL, Teyler TJ (2001). Electrical stimuli patterned after the theta-rhythm induce multiple forms of LTP.. J Neurophysiol.

[26] Hagan JJ, Salamone JD, Simpson J, Iversen SD, Morris RG (1988). Place navigation in rats is impaired by lesions of medial septum and diagonal band but not nucleus basalis magnocellularis.. Behav Brain Res.

[27] Hepler DJ, Olton DS, Wenk GL, Coyle JT (1985). Lesions in nucleus basalis magnocellularis and medial septal area of rats produce qualitatively similar memory impairments.. J Neurosci.

[28] Kelsey JE, Landry BA (1988). Medial septal lesions disrupt spatial mapping ability in rats.. Behav Neurosci.

[29] M'Harzi M, Jarrard LE (1992). Effects of medial and lateral septal lesions on acquisition of a place and cue radial maze task.. Behav Brain Res.

[30] Miyamoto M, Kato J, Narumi S, Nagaoka A (1987). Characteristics of memory impairment following lesioning of the basal forebrain and medial septal nucleus in rats.. Brain Res.

[31] Mizumori SJ, Perez GM, Alvarado MC, Barnes CA, McNaughton BL (1990). Reversible inactivation of the medial septum differentially affects two forms of learning in rats.. Brain Res.

[32] Baxter MG, Bucci DJ, Gorman LK, Wiley RG, Gallagher M (1995). Selective immunotoxic lesions of basal forebrain cholinergic cells: effects on learning and memory in rats.. Behav Neurosci.

[33] Baxter MG, Gallagher M (1996). Intact spatial learning in both young and aged rats following selective removal of hippocampal cholinergic input.. Behav Neurosci.

[34] Frielingsdorf H, Thal LJ, Pizzo DP (2006). The septohippocampal cholinergic system and spatial working memory in the Morris water maze.. Behav Brain Res.

[35] Gibbs RB, Johnson DA (2007). Cholinergic lesions produce task-selective effects on delayed matching to position and configural association learning related to response pattern and strategy.. Neurobiol Learn Mem.

[36] Jonasson Z, Cahill JF, Tobey RE, Baxter MG (2004). Sexually dimorphic effects of hippocampal cholinergic deafferentation in rats.. Eur J Neurosci.

[37] Kirby BP, Rawlins JN (2003). The role of the septo-hippocampal cholinergic projection in T-maze rewarded alternation.. Behav Brain Res.

[38] McMahan RW, Sobel TJ, Baxter MG (1997). Selective immunolesions of hippocampal cholinergic input fail to impair spatial working memory.. Hippocampus.

[39] Winters BD, Dunnett SB (2004). Selective lesioning of the cholinergic septo-hippocampal pathway does not disrupt spatial short-term memory: a comparison with the effects of fimbria-fornix lesions.. Behav Neurosci.

[40] Fletcher BR, Baxter MG, Guzowski JF, Shapiro ML, Rapp PR (2007). Selective cholinergic depletion of the hippocampus spares both behaviorally induced Arc transcription and spatial learning and memory.. Hippocampus.

[41] Shen J, Barnes CA, Wenk GL, McNaughton BL (1996). Differential effects of selective immunotoxic lesions of medial septal cholinergic cells on spatial working and reference memory.. Behav Neurosci.

[42] van der Staay FJ, Bouger P, Lehmann O, Lazarus C, Cosquer B (2006). Long-term effects of immunotoxic cholinergic lesions in the septum on acquisition of the cone-field task and noncognitive measures in rats.. Hippocampus.

[43] Fitz NF, Gibbs RB, Johnson DA (2008). Selective lesion of septal cholinergic neurons in rats impairs acquisition of a delayed matching to position T-maze task by delaying the shift from a response to a place strategy.. Brain Res Bull.

[44] Johnson DA, Zambon NJ, Gibbs RB (2002). Selective lesion of cholinergic neurons in the medial septum by 192 IgG-saporin impairs learning in a delayed matching to position T-maze paradigm.. Brain Res.

[45] Janis LS, Glasier MM, Fulop Z, Stein DG (1998). Intraseptal injections of 192 IgG saporin produce deficits for strategy selection in spatial-memory tasks.. Behav Brain Res.

[46] Berger-Sweeney J, Heckers S, Mesulam MM, Wiley RG, Lappi DA (1994). Differential effects on spatial navigation of immunotoxin-induced cholinergic lesions of the medial septal area and nucleus basalis magnocellularis.. J Neurosci.

[47] Walsh TJ, Herzog CD, Gandhi C, Stackman RW, Wiley RG (1996). Injection of IgG 192-saporin into the medial septum produces cholinergic hypofunction and dose-dependent working memory deficits.. Brain Res.

[48] Lehmann O, Grottick AJ, Cassel JC, Higgins GA (2003). A double dissociation between serial reaction time and radial maze performance in rats subjected to 192 IgG-saporin lesions of the nucleus basalis and/or the septal region.. Eur J Neurosci.

[49] Huber KM, Mauk MD, Kelly PT (1995). Distinct LTP induction mechanisms: contribution of NMDA receptors and voltage-dependent calcium channels.. J Neurophysiol.

[50] Lauterborn JC, Rex CS, Kramar E, Chen LY, Pandyarajan V (2007). Brain-derived neurotrophic factor rescues synaptic plasticity in a mouse model of fragile X syndrome.. J Neurosci.

[51] Cobb SR, Davies CH (2005). Cholinergic modulation of hippocampal cells and circuits.. J Physiol.

[52] Radcliffe KA, Fisher JL, Gray R, Dani JA (1999). Nicotinic modulation of glutamate and GABA synaptic transmission of hippocampal neurons.. Ann N Y Acad Sci.

[53] Muller D, Joly M, Lynch G (1988). Contributions of quisqualate and NMDA receptors to the induction and expression of LTP.. Science.

[54] Robinson HP, Kawai N (1993). Single channel properties at the synaptic site.. Exs.

[55] Tang CM, Shi QY, Katchman A, Lynch G (1991). Modulation of the time course of fast EPSCs and glutamate channel kinetics by aniracetam.. Science.

[56] Vaithianathan T, Manivannan K, Kleene R, Bahr BA, Dey MP (2005). Single channel recordings from synaptosomal AMPA receptors.. Cell Biochem Biophys.

[57] Mayer ML, Westbrook GL (1987). Permeation and block of N-methyl-D-aspartic acid receptor channels by divalent cations in mouse cultured central neurones.. J Physiol.

[58] Pizzo DP, Waite JJ, Thal LJ, Winkler J (1999). Intraparenchymal infusions of 192 IgG-saporin: development of a method for selective and discrete lesioning of cholinergic basal forebrain nuclei.. J Neurosci Methods.

[59] Yoder RM, Pang KC (2005). Involvement of GABAergic and cholinergic medial septal neurons in hippocampal theta rhythm.. Hippocampus.

[60] Martin MM, Wallace DG (2007). Selective hippocampal cholinergic deafferentation impairs self-movement cue use during a food hoarding task.. Behav Brain Res.

[61] Monteiro Moreira K, Lima Ferreira T, Vecchio Fornari R, Perez Figueredo LZ, Menezes Oliveira MG (2005). Interaction between M1-muscarinic and glutamatergic NMDA receptors on an inhibitory avoidance task.. Brain Res Bull.

[62] Ohno M, Watanabe S (1996). Interactive processing between glutamatergic and cholinergic systems involved in inhibitory avoidance learning of rats.. Eur J Pharmacol.

[63] Ciamei A, Aversano M, Cestari V, Castellano C (2001). Effects of MK-801 and nicotine combinations on memory consolidation in CD1 mice.. Psychopharmacology (Berl).

[64] Gould TJ, Lewis MC (2005). Coantagonism of glutamate receptors and nicotinic acetylcholinergic receptors disrupts fear conditioning and latent inhibition of fear conditioning.. Learn Mem.

[65] Levin ED, Bettegowda C, Weaver T, Christopher NC (1998). Nicotine-dizocilpine interactions and working and reference memory performance of rats in the radial-arm maze.. Pharmacol Biochem Behav.

[66] Tsukada H, Miyasato K, Nishiyama S, Fukumoto D, Kakiuchi T (2005). Nicotine normalizes increased prefrontal cortical dopamine D1 receptor binding and decreased working memory performance produced by repeated pretreatment with MK-801: a PET study in conscious monkeys.. Neuropsychopharmacology.

[67] Figueredo LZ, Moreira KM, Ferreira TL, Fornari RV, Oliveira MG (2008). Interaction between glutamatergic-NMDA and cholinergic-muscarinic systems in classical fear conditioning.. Brain Res Bull.

[68] Jafari-Sabet M (2006). NMDA receptor blockers prevents the facilitatory effects of post-training intra-dorsal hippocampal NMDA and physostigmine on memory retention of passive avoidance learning in rats.. Behav Brain Res.

[69] Rezayof A, Shirazi-Zand Z, Zarrindast MR, Nayer-Nouri T (2010). Nicotine improves ethanol-induced memory impairment: the role of dorsal hippocampal NMDA receptors.. Life Sci.

[70] Levin ED, Sledge D, Baruah A, Addy NA (2003). Ventral hippocampal NMDA blockade and nicotinic effects on memory function.. Brain Res Bull.

[71] Andre JM, Leach PT, Gould TJ (2011). Nicotine ameliorates NMDA receptor antagonist-induced deficits in contextual fear conditioning through high-affinity nicotinic acetylcholine receptors in the hippocampus.. Neuropharmacology.

[72] Aigner TG (1995). Pharmacology of memory: cholinergic-glutamatergic interactions.. Curr Opin Neurobiol.

[73] Ovsepian SV, Anwyl R, Rowan MJ (2004). Endogenous acetylcholine lowers the threshold for long-term potentiation induction in the CA1 area through muscarinic receptor activation: in vivo study.. Eur J Neurosci.

[74] Blokland A (1995). Acetylcholine: a neurotransmitter for learning and memory?. Brain Res Brain Res Rev.

[75] Hasselmo ME (1999). Neuromodulation: acetylcholine and memory consolidation.. Trends Cogn Sci.

[76] Shinoe T, Matsui M, Taketo MM, Manabe T (2005). Modulation of synaptic plasticity by physiological activation of M1 muscarinic acetylcholine receptors in the mouse hippocampus.. J Neurosci.

[77] Auerbach JM, Segal M (1996). Muscarinic receptors mediating depression and long-term potentiation in rat hippocampus.. J Physiol.

[78] Fernandez de Sevilla D, Nunez A, Borde M, Malinow R, Buno W (2008). Cholinergic-mediated IP3-receptor activation induces long-lasting synaptic enhancement in CA1 pyramidal neurons.. J Neurosci.

[79] Kuczewski N, Aztiria E, Leanza G, Domenici L (2005). Selective cholinergic immunolesioning affects synaptic plasticity in developing visual cortex.. Eur J Neurosci.

[80] Birthelmer A, Ehret A, Amtage F, Forster S, Lehmann O (2003). Neurotransmitter release and its presynaptic modulation in the rat hippocampus after selective damage to cholinergic or/and serotonergic afferents.. Brain Res Bull.

[81] Yamazaki Y, Hamaue N, Sumikawa K (2002). Nicotine compensates for the loss of cholinergic function to enhance long-term potentiation induction.. Brain Res.

[82] Vaglenova J, Parameshwaran K, Suppiramaniam V, Breese CR, Pandiella N (2008). Long-lasting teratogenic effects of nicotine on cognition: gender specificity and role of AMPA receptor function.. Neurobiol Learn Mem.

[83] Yamazaki Y, Jia Y, Niu R, Sumikawa K (2006). Nicotine exposure in vivo induces long-lasting enhancement of NMDA receptor-mediated currents in the hippocampus.. Eur J Neurosci.

[84] Pitler TA, Alger BE (1992). Cholinergic excitation of GABAergic interneurons in the rat hippocampal slice.. J Physiol.

[85] Jouvenceau A, Billard JM, Wiley RG, Lamour Y, Dutar P (1994). Cholinergic denervation of the rat hippocampus by 192-IgG-saporin: electrophysiological evidence.. Neuroreport.

[86] Jouvenceau A, Billard JM, Lamour Y, Dutar P (1997). Potentiation of glutamatergic EPSPs in rat CA1 hippocampal neurons after selective cholinergic denervation by 192 IgG-saporin.. Synapse.

[87] Hefft S, Hulo S, Bertrand D, Muller D (1999). Synaptic transmission at nicotinic acetylcholine receptors in rat hippocampal organotypic cultures and slices.. J Physiol.

[88] Kanju PM, Parameshwaran K, Sims C, Bahr BA, Shonesy BC (2008). Ampakine CX516 ameliorates functional deficits in AMPA receptors in a hippocampal slice model of protein accumulation.. Exp Neurol.

[89] Salter MW, Dong Y, Kalia LV, Liu XJ, Pitcher G, Van Dongen AM (2009). Regulation of NMDA Receptors by Kinases and Phosphatases.. Biology of the NMDA Receptor.

[90] Lin Y, Jover-Mengual T, Wong J, Bennett MV, Zukin RS (2006). PSD-95 and PKC converge in regulating NMDA receptor trafficking and gating.. Proc Natl Acad Sci U S A.

[91] Xu F, Plummer MR, Len GW, Nakazawa T, Yamamoto T (2006). Brain-derived neurotrophic factor rapidly increases NMDA receptor channel activity through Fyn-mediated phosphorylation.. Brain Res.

[92] Kristensen AS, Jenkins MA, Banke TG, Schousboe A, Makino Y (2011). Mechanism of Ca2+/calmodulin-dependent kinase II regulation of AMPA receptor gating.. Nat Neurosci.

[93] Banke TG, Bowie D, Lee H, Huganir RL, Schousboe A (2000). Control of GluR1 AMPA receptor function by cAMP-dependent protein kinase.. J Neurosci.

[94] Derkach V, Barria A, Soderling TR (1999). Ca2+/calmodulin-kinase II enhances channel conductance of alpha-amino-3-hydroxy-5-methyl-4-isoxazolepropionate type glutamate receptors.. Proc Natl Acad Sci U S A.

[95] Kamsler A, McHugh TJ, Gerber D, Huang SY, Tonegawa S (2010). Presynaptic m1 muscarinic receptors are necessary for mGluR long-term depression in the hippocampus.. Proc Natl Acad Sci U S A.

[96] Lim CS, Hwang YK, Kim D, Cho SH, Banuelos C (2011). Increased interactions between PKA and NF-kappaB signaling in the hippocampus following loss of cholinergic input.. Neuroscience.

[97] Givens B, Olton DS (1995). Bidirectional modulation of scopolamine-induced working memory impairments by muscarinic activation of the medial septal area.. Neurobiol Learn Mem.

[98] Markowska AL, Olton DS, Givens B (1995). Cholinergic manipulations in the medial septal area: age-related effects on working memory and hippocampal electrophysiology.. J Neurosci.

[99] Stewart M, Fox SE (1990). Do septal neurons pace the hippocampal theta rhythm?. Trends Neurosci.

[100] Paxinos G, Watson C (1998). The Rat Brain in Stereotaxic Coordinates.

[101] Johnson MW, Chotiner JK, Watson JB (1997). Isolation and characterization of synaptoneurosomes from single rat hippocampal slices.. J Neurosci Methods.

